# Significance of continuing audiovestibular practice during Covid-19 pandemic

**DOI:** 10.1186/s43163-021-00073-1

**Published:** 2021-01-25

**Authors:** Oğuz Yilmaz, Dilara Bayazit, Handan Yaman, Berna Özge Mutlu

**Affiliations:** Department of Audiology, Istanbul Medipol Mega University Hospital, Göztepe Mah. Metin Sk. Nu:4, Bağcılar, Istanbul, Turkey

**Keywords:** Covid-19, SARS-CoV-2, Audiology, Vestibular

## Abstract

**Background:**

We aimed to evaluate the significance of continuing audiovestibular practice during the Covid-19 pandemic in the audiology clinic of the university hospital.

**Methods:**

The precautions, test procedures, and risk factors associated with the practice of audiology were evaluated. The number and diagnoses of the cases between 23 March and 23 May 2020 were also compared with the results of the same 2 months in 2019 in an attempt to evaluate the alterations in the audiology practice.

**Results:**

The audiology practice has continued during the pandemic, and numerous audiovestibular tests like newborn hearing screening, pure tone and speech audiometry, auditory brainstem response (ABR) test, bedside vestibular assessment, videomystagmography (VNG), caloric test, vestibular evoked myogenic potential (VEMP), computerized dynamic posturography (CDP), video head impulse test (vHIT), intraoperative cochlear implant measurement, and postop cochlear implant fittings were performed. The number of tests has decreased significantly in the course of the pandemic (*p* < 0.01). No evidence of Covid-19 disease was detected in the audiology staff and patients. In general, no major risk was seen during testing under protective measures except for vestibular testing which induced vomiting and taking out the mask for lip reading during cochlear implant fitting in the elderly.

**Conclusion:**

It is possible to perform audiovestibular tests during the Covid-19 pandemic by wearing necessary protective equipment and disinfecting the potential surfaces. Vomiting during vestibular tests, uncovering the nose and mouth for lip reading, and small-sized test cabins are the main risk factors of contamination in the audiology clinics.

## Bullet point summary

The audiological test protocols of the hospital are presented.

Covid-19 pandemic has a negative impact on audiological services.

Vomiting and communication with lip reading may increase the risk of contamination.

The main risk factors and protection methods for possible pandemics in the future are defined.

## Background

Since December 2019, when SARS-CoV-2 first emerged in Wuhan, China, coronavirus disease (Covid-19) has been a major world health problem in the form of a pandemic.

The risk of infection for health care professionals and patients in audiology laboratories is important. There is no vaccine or treatment for now. Social distancing or reducing interpersonal and community interaction is the main way to minimize disease transmission [1].

It is still unclear or controversial whether it is safe to perform elective audiological tests. Despite the decrease in the new cases of Covid-19, most of the audiology clinics still hesitate to perform elective audiological tests with concerns related to the safety of patients and physicians.

In this study, we aimed to evaluate the significance of continuing audiovestibular practice during the Covid-19 pandemic.

## Methods

During the Covid-19 pandemic, between 23 March and 23 May 2020, the working protocol of the audiovestibular clinic of the university was evaluated. As a general regulation, at the entrance of the hospital, body temperatures of health care professionals, patients, and family members have been monitored using an infrared thermometer. Those who had a body temperature above 37.3 °C9 were referred to Covid-19 clinic of the same hospital.

Everybody entering the hospital including the hospital staff had to wear a surgical mask. In the waiting area of the audiology clinic, the rule of social distancing was applied. In the audiology clinic, in addition to the audiologist, only one of the family members was allowed to accompany a child being tested. No companion was allowed to enter the test room while testing an adult patient.

As a precaution, the number of audiology personnel was reduced by switching to the shift system. All the personnel had protective tools like masks (surgical or N95 type), gloves, and face shields. Hand washing is instructed after every test. Alcohol-based disinfectants were used for hand hygiene and cleansing the audiovestibular devices. All test devices and the contact surfaces in the rooms were cleaned with the same disinfectant 3 times a day. Disposable or sterilized electrodes or probes were used, if applicable. Otherwise, the equipment or cables were cleaned using disinfectants. The test interval between the patients was set to half an hour.

In newborn hearing screening, under normal circumstances, detection and intervention programs in Turkey have 24 h and first, third, and sixth month benchmarks. The newborn hearing screening of the babies who were born in the same hospital was done, and they are discharged. If the newborn was admitted to the neonatal intensive care unit or could not pass three successive hearing screenings, auditory brain stem response (ABR) and the other test batteries were performed. During the Covid-19 pandemic, the Ministry of Health restricted the performance of second hearing screening and clinical ABR testing. However, newborn hearing screening was continued.

On pure tone and speech audiometry, no trouble happened while putting on headphones and bone vibrators in the presence of surgical masks to the patients. The audiologist had to take out the mask during speech tests. Sterile probes were used for each patient on tympanometry.

On ABR testing, the audiologist wore an N95 mask due to the small size of and absence of the ventilator in the test room and a long test procedure of 1 to 2 h.

Regarding the cochlear implants (CI), since the patients had negative PCR test result for Covid-19, and normal laboratory and radiological findings, the intraoperative measurements of CIs were done wearing N95 masks in the operation rooms. Postoperative CI fitting was done under the aforementioned protocols unless lip reading was necessary, in which the mask was taken out.

Vestibular tests were done under the aforementioned protocols. The goggle glasses were disinfected with alcohol-based disinfectants. The mask of the patient was taken out for a while in order to place the electrodes during vestibular evoked myogenic potentials (VEMP) testing. Water caloric, video head impulse test (vHIT), videonystagmography (VNG), and computerized dynamic posturography (CDP) were performed.

## Results

During the Covid-19 pandemic, a significant decrease was encountered in the number of tests performed in the audiology clinic as a result of governmental regulations, social isolation rules, and hesitation of the patients to visit the audiology clinic in the hospital (*p* < 0.01) (Table 1).

**Table 1:** The audiology clinic test numbers of 1-year interval during the Covid-19 pandemic

Tests performed in the audiology clinic	Period 23 May to 23 March 2019, no. of cases	Period 23 May to 23 March 2020, no. of cases	Alteration between the periods, (%)
Audiometry	657	128	81
Immitancemetry	529	96	82
ABR	47	11	77
A-ABR	419	124	70
VEMP	31	3	90
VNG	48	9	81
Caloric test	17	9	47
vHIT	14	1	93
CDP	6	0	100
Play audiometry	22	0	100
OAE	17	2	88
Tinnitus assessment	2	7	250
Intraop CI	5	2	60
CI fitting	7	3	57

*ABR* auditory brainstem response testing, *A-ABR* automated ABR, *VEMP* vestibular evoked myogenic potential, *VNG* videonystagmography, *vHIT* video head impulse test, *CDP* computerized dynamic posturography, *OAE* optoacoustic emission testing, *Intraop CI* intraoperative cochlear implant measurement

Under normal circumstances, there were 5 audiologists and 2 audiometrists in the audiology clinic. During the period of the pandemic, 1 audiologist and 1 audiometrist worked in the clinic. All patients who were tested in the audiology department were seen by an ENT physician except for the newborns who were seen by pediatricians.

No evidence of Covid-19 disease was detected in the audiology staff as evidenced by their negative PCR testing and the absence of signs and symptoms of the disease. None of the patients was suspected of having Covid-19 according to history and absence of signs and symptoms of the disease like high fever and cough.

In general, no major risk was seen during testing under protective measures. However, one of the major risks encountered was during vestibular testing, in which 3 patients vomited during VNG tests. During CI fitting of the elderly patients, the audiologist had to take out the mask in order to overcome communication problems through lip reading.

## Discussion

Covid-19 is an acute respiratory illness caused by SARS-CoV-2 infection [2]. This pandemic is a global public health emergency affecting millions of people worldwide.

Although the majority of people postponed their treatment during the pandemic, still, there are people who seek treatment or screening based on audiological measurements. Safety is critical not only for patients, but also for audiologists. It is essential to take the necessary precautions to protect both of them while performing the audiovestibular tests. At this point, the results of our study may be important.

Overall, a decrease of almost 80% was encountered in the number of audiology tests within one during the Covid-19 pandemic. Governmental regulations, social isolation rules, and patient reservations might be associated with this decreases to go to medical centers. However, a significant increase was encountered in the number of patients who applied for tinnitus management. Emotional impact of the pandemic might have played a role in this matter [3, 4].

In our experience, there are several risk factors associated with contamination in an audiology setting. First, the test cabins in audiology departments are small in size. The patient and audiologist will have to talk to each other in a short distance, which may cause contamination potentially by virus-loaded droplets. This is mainly true in speech audiometry areas and when lip reading is needed. Speech and the possibility of sneezing may lead to contamination or dissemination of infection [5].

Surgical masks can be protective against droplets. However, N95 masks should be used when the likelihood of Covid-19 infection increases as in the intensive care units and when there is close contact with a Covid-19-positive person. In the study, the optical sizer intake port was placed 15 cm away from the nares. The distance specified in the study is considered to be similar to the microphone distance from the mouth used in speech audiometry. Accordingly, surgical masks can facilitate sufficient protection unless the audiologist or patient sneezes during the test. If one of them sneezes, it is recommended to disinfect the room and microphone unless the sneezer was wearing an N95 mask. It should be remembered that a surgical mask acts as a low pass filter and leads to 12 dB HL change at speech reception test (SRT) [6]. The overall effect of a mask on a patient with hearing impairment will be more significant [7].

The second major risk is related with vomiting of the patient during vestibular tests. Vomiting may be accompanied by forced breathing, both of which can increase the risk of contamination [8]. It is suggested that patients may have gastrointestinal involvement due to the presence of Covid-19 virus particles in the stool [9], and this may cause possible transmission with vomiting, although this issue needs confirmation.

There is a risk of transmission through audiological equipment. There are some studies related to the viability of viral particles on inert surfaces [10]. Although the transmission risk is still unknown, the frequently touched surfaces should be cleaned and disinfected. These surfaces are desks, door handles, light switches, counters, handles, desks, phones, keyboards, toilets, taps, and sinks. We applied disinfection to the areas in our clinic 3 times a day [11].

World Health Organization (WHO) and national guides can be used for general considerations [12]. But the audiology associations guidelines specific for the audiology clinics are also very helpful [13–15].

## Conclusion

It is possible to perform audiovestibular tests during the Covid-19 pandemic by wearing necessary protective equipment and disinfecting the potential surfaces. Vomiting during vestibular tests, uncovering the nose and mouth for lip reading, and small-sized test cabins are the main risk factors of contamination in the audiology clinics.

## Data Availability

Not applicable.

## Abbreviations

ABR: Auditory brainstem response
CDP: Computerized dynamic posturography
CI: Cochlear implant
dB: Decibel
ENT: Ear, nose, throat
HL: Hearing level
PCR: Polymerase chain reaction
SRT: Speech reception test
VEMP: Vestibular evoked myogenic potentials
vHIT: Video head impulse test
VNG: Videonystagmography
WHO: World Health Organization
